# Hydroxysafflor Yellow A Suppresses Platelet Activating Factor-Induced Activation of Human Small Airway Epithelial Cells

**DOI:** 10.3389/fphar.2018.00859

**Published:** 2018-08-03

**Authors:** Xinjing Guo, Meng Zheng, Ruiyan Pan, Baoxia Zang, Ming Jin

**Affiliations:** Department of Pharmacology, Beijing Anzhen Hospital, Capital Medical University, Beijing Institute of Heart Lung and Blood Vessel Diseases, Beijing, China

**Keywords:** hydroxysafflor yellow A, asthma, platelet activating factor, human small airway epithelial cell, inflammation

## Abstract

Hydroxysafflor yellow A (HSYA) is a chemical component isolated from the Chinese medicine *Carthamus tinctorius* L. HSYA has numerous pharmacological effects, including protecting against and mitigating some respiratory diseases such as acute lung injury and chronic obstructive pulmonary disease; however, its effect on asthma remains unclear. We previously found that HSYA attenuated ovalbumin-induced allergic asthma in guinea pigs. Platelet activating factor (PAF) is a phospholipid mediator of inflammation and an important factor in the pathological process of asthma. In this study, we investigated the anti-inflammatory effects of HSYA and its underlying mechanisms in PAF-induced human small airway epithelial cells (HSAECs). PAF-activated cells were pretreated with HSYA and/or the PAF receptor inhibitor, ginkgolide B, and we observed changes in the expression of interleukin (IL)-1β, IL-6, and tumor necrosis factor alpha, monolayer permeability of HSAECs, and inflammatory signaling pathways. HSYA attenuated the PAF-induced increase in expression of inflammatory factors and destruction of cell-barrier function, and inhibited the expression of protein kinase C, mitogen-activated protein kinases, activator protein-1, and nuclear factor-κB activation induced by PAF. These findings suggest that HSYA may represent a potential new drug for the treatment of asthma.

## Introduction

Bronchial asthma (asthma) is a chronic inflammatory airway disease characterized by allergic airway inflammation, airway remodeling, and airway hyperresponsiveness (AHR) (Liu et al., 2017). More than 300 million people worldwide suffer from asthma (Al-Busaidi et al., 2015), and its prevalence and mortality are continuing to increase. The main pathological change in asthma involves chronic inflammatory injury, which is also the core feature of an asthma attack. Inhibiting the inflammatory response is thus an important means of relieving symptoms in patients with asthmatic diseases (Chung, 1986; Locksley, 2010; Kim et al., 2013).

Airway epithelial cells provide the first barrier against the outside environment and thus play a central role in the initiation and development of airway inflammation in asthma (Hammad and Lambrecht, 2015). Following stimulation by inflammatory factors, airway epithelial cells can increase the expression of various growth factors and synthesis of the extracellular matrix, and induce further changes in airway remodeling, such as smooth muscle cell proliferation and neovascularization (Erle and Sheppard, 2014; Georas and Rezaee, 2014). The occurrence and development of asthma inflammation involve a large number of inflammatory mediators, including platelet activating factor (PAF), which plays an important role in the pathological process of asthma. Previous animal studies found that PAF injection caused various types of asthma symptoms, such as bronchial constriction, AHR, and inflammatory cell infiltration. PAF levels in bronchial lavage fluid and plasma were significantly elevated in asthmatic patients compared with normal subjects (Lou et al., 1998; Kasperska-Zajac et al., 2008a,b). Binding of PAF to its receptor triggers inflammatory downstream signal transduction, including activation of protein kinase C (PKC), causing an increase in cellular calcium levels. PAF can also activate signal pathways such as nuclear factor-κB (NFκB) and mitogen-activated protein kinases (MAPK) (Lukashova et al., 2001), and enhance the transcriptional activity of activator protein-1 (AP1), which aggravates the symptoms of asthma (McLaughlin et al., 2006; Das et al., 2016). Inhibiting the PAF-induced inflammatory response thus represents an important means of relieving AHR and treating asthma.

Despite recent progress in the pharmacological treatment of asthma, there is still no ideal medical treatment. Asthma symptoms can usually be controlled using corticosteroids and long-acting β-agonists, and new biologics such as omalizumab (anti-IgE antibody), dupilumab (anti-IL-4Rα antibody), and mepolizumab (anti-IL-5) are also showing promise (Hansbro et al., 2011, 2013). However, current asthma drugs may not reverse or delay airway remodeling, and some hormones have obvious side effects. New treatments, especially non-hormonal drugs, are thus required to treat asthma patients. Traditional Chinese medicines have recently attracted increasing attention in relation to protecting against inflammation, with promising potential for asthma.

Hydroxysafflor yellow A (HSYA) is a chemical component isolated from the Chinese medicine *Carthamus tinctorius* L., with numerous pharmacological effects. It is a traditionally used for promoting blood circulation and preventing clotting. It is also an effective anti-inflammatory (Song et al., 2013; Cheng et al., 2016) and anti-oxidant agent (Sun et al., 2012), and well as demonstrating other cardio-cerebrovascular pharmacological effects (Nie et al., 2012). HSYA has shown strong protective and mitigation effects in acute lung injury, chronic obstructive pulmonary disease, and pulmonary hypertension (Liu et al., 2014; Jin et al., 2016; Li et al., 2016). However, the mechanisms of HSYA in treating airway inflammation in asthma remain unclear.

In this study, we investigated the inhibitory effect of HSYA on PAF-induced asthma-related inflammation in human small airway epithelial cells (HSAECs), and explored the underlying mechanisms. These results will support further development of HSYA as a novel anti-asthma drug.

## Materials and Methods

### Chemicals and Reagents

Platelet activating factor was obtained from Merck (Darmstadt, Germany) and the working PAF solution was freshly prepared with anhydrous dimethylsulfoxide (DMSO). Antibodies against phospho-inhibitor of nuclear factor κB (IκBα), IκBα, ERK, phospho-ERK, p38, phospho-p38, JNK, phospho-JNK, phospho-c-Jun, and β-actin were purchased from Cell Signaling Technology (Danvers, MA, United States). The PKC antibody was produced by Abcam (Cambridge, United Kingdom). Enzyme-linked immunosorbent assay (ELISA) kits were purchased from Shanghai Xitang Biotechnology Co., Ltd. (Shanghai, China). The Protease phosphatase inhibitor mixtures, Fluo-3 AM, and GAPDH antibody were from Beyotime Institute of Biotechnology (Jiangsu, China). The PAF receptor inhibitor, ginkgolide B (GB) (purity was 99.49), was from Chengdu Mansite Biotechnology (Chengdu, China). TRIzol reagent was from Invitrogen (Carlsbad, CA, United States), and the SYBR Premix ExTaq^TM^ (Perfect Real Time) kit was from Agilent Technologies (Santa Clara, CA, United States). Hank’s balanced salt solution and phosphate-buffered solution (PBS) were produced by Solarbio (Beijing, China).

### Cell Culture

HSAEC1-KT HSAECs were purchased from the American Type Culture Collection (Manassas, VA, United States). Cells were maintained in Dulbecco’s Modified Eagle Medium (DMEM; high glucose) containing 10% fetal bovine serum with 1% penicillin–streptomycin in a 5% CO_2_ atmosphere at 37°C. After incubation for 24 h to reach 80–90% confluence and starvation for 12 h in serum-free DMEM, the cells were pretreated with or without HSYA at various concentrations, or with 10^-7^ mol/L GB (diluted in anhydrous DMSO) for 30 min, and then incubated with or without PAF for a fixed time. Equivalent volumes of solvent instead of the various drugs were added to each group of cells as controls.

### Preparation of HSYA

**FIGURE 1:**
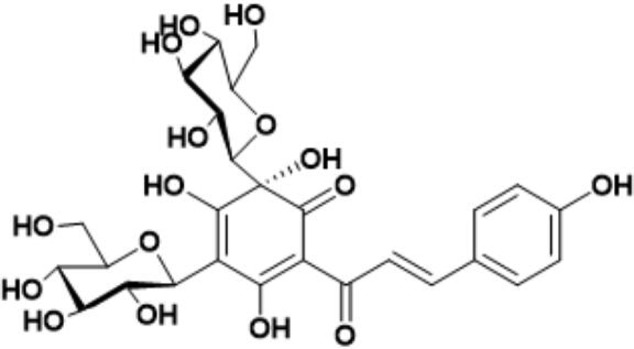
Molecular structure of HSYA.

**FIGURE 2:**
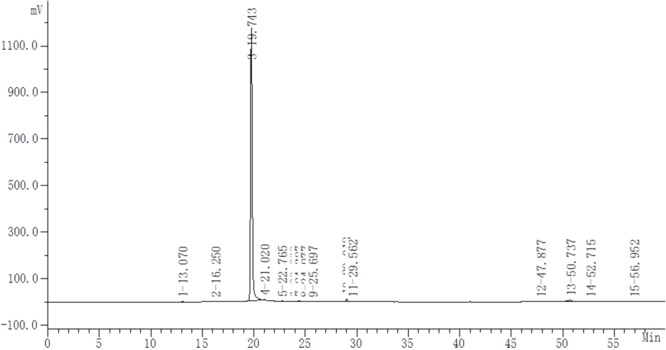
High-performance liquid chromatography analysis of purified HSYA. The absorbance was measured at 405 nm (20-min peak is HSYA).

Safflower is the dry flower of *C. tinctorius* L., which was grown in Tacheng, Xinjiang Uygur Autonomous Region, China. It was obtained from Huahui Kaide Pharmaceutical Co., Ltd. (Shanxi, China) and identified by Professor Jiashi Li (Beijing University of Traditional Chinese Medicine). Our research group has the technology and experience for separating and extracting HSYA. HSYA was isolated and purified from the aqueous extract of *C. tinctorius* L. by macroporous resin-gel column chromatography, as described previously (Zang et al., 2008). The molecular weight of HSYA is 612 and its molecular structure is presented in **Figure 1** (Feng et al., 2013). HSYA was analyzed by high-performance liquid chromatography (Wang et al., 2014) and the purity was 95.2%, determined by the area normalization method (**Figure 2**). The prepared HSYA was dissolved in DMEM for subsequent experiments.

### Real-Time Polymerase Chain Reaction (RT-PCR)

During early inflammation, PAF can induce airway epithelial cells to secrete many inflammatory factors and thus promote asthma-related injuries. We assessed the effects of HSYA on PAF-induced mRNA expression levels of interleukin (IL)-1β, IL-6, and tumor necrosis factor alpha (TNF-α). HSAECs were plated in six-well plates and incubated for 24 h, followed by starvation for 12 h. The cells were then divided into eight groups as follows: normal control; HSYA blank control (81 μmol/L); PAF (10^-7^ μmol/L); PAF + HSYA (9 μmol/L); PAF + HSYA (27 μmol/L); PAF + HSYA (81 μmol/L); PAF + GB (10^-7^ μmol/L); and PAF + GB + HSYA (81 μmol/L). Cells in the PAF + GB group received an equivalent volume of DMEM instead of HSYA; cells in the PAF + HSYA (9, 27, and 81 μmol/L) groups received an equivalent volume of DMSO instead of GB; and cells in the PAF group received equivalent volumes of DMEM and DMSO instead of HSYA and GB, respectively. Cells in the HSYA blank control group received equivalent volumes of DMSO instead of PAF and GB, and cells in the normal control group received an equivalent volume of DMEM instead of HSYA, and equivalent volumes of DMSO instead of PAF and GB. The concentration of DMSO in the culture medium in each experimental group was 4.23 × 10^-9^ mL/mL. We pretreated cells with or without various concentrations of HSYA or GB (10^-7^ mol/L) for 30 min, followed by the addition of PAF (10^-7^ mol/L) to each well and incubation for 4 h. We then isolated total RNA from HSAECs using TRIzol reagent (Invitrogen), according to the manufacturer’s protocol, and detected the concentration and quality of RNA using a NanoDrop 2000 device (Thermo Scientific, Wilmington, DE, United States). First-strand cDNA synthesis was performed using a reverse transcriptase kit (Promega) with 2 μg RNA. mRNA levels of the genes were measured by RT-PCR using a SYBR^®^ Premix ExTaq^TM^ kit, under the following conditions: 10 min at 95°C for initial denaturation, followed by 39 cycles of 95°C for 10 s, 55°C for 30 s, and finally 55–95°C in increments of 0.5°C every 10 s for melting curve analysis. The primer sequences were as follows: IL-6, forward 5′-GTGAAAGCAGCAAAGAGGC-3′ and reverse 5′-CATTTGTGGTTGGGTCAGG-3′; IL-1β, forward 5′-TACGAATCTCCGACCACCACTACAG-3′ and reverse 5′-TGGAGGTGGAGAGCTTTCAGTTCATATG-3′; TNF-α, forward 5′-CTCCACCCATGTGCTCCTCAC-3′ and reverse 5′-CCCAAAGTAGACCTGCCCAGA-3′; and GAPDH, forward 5′-CCATGAGAAGTATGACAACAGCC-3′ and reverse 5′-GGGTGCTAAGCAGTTGGTG-3′. Relative quantification was assayed using the 2^–∆∆Ct^ method (Livak and Schmittgen, 2001) with data normalized to GAPDH.

### ELISA

We detected inflammatory cytokines in HSAECs using ELISA kits to evaluate the effects of HSYA on IL-6, IL-1β, and TNF-α protein expression levels induced by PAF. Cells were seeded in six-well plates, cultured in a 5% CO_2_ atmosphere at 37°C for 24 h, and then starved for 12 h. Cells were pretreated as above, followed by incubation with PAF (10^-7^ μmol/L) for 12 h. The culture medium was collected and the concentrations of the inflammatory cytokines in the medium were measured according to the manufacturer’s protocol (Shanghai Xitang Biotechnology Co., Ltd.).

### Determination of Epithelial Monolayer Permeability

HSAECs were seeded at 1 × 10^5^/cm^2^ onto a microporous membrane in the top chambers of Transwell^®^-24-well permeable supports (Corning Costar, NY, United States), above a substrate to collect solution permeating from the top side. When the cells had grown and fused to form monolayers, they were starved for 12 h and then pretreated as above, followed by stimulation with PAF (10^-7^ μmol/L) for 24 h. The pretreated cells were carefully washed three times with Hank’s balanced salt solution (pH 7.4), incubated at 37°C for 30 min, and the Hank’s balanced salt solution in the wells was then aspirated to prevent interference. Lucifer yellow 40 μg/mL (Merck, Darmstadt, Germany) was then added to the top chamber and the substrate-side liquid was collected after 1 h incubation at 37°C. The absorbance was measured at an excitation wavelength of 427 nm and emission wavelength of 536 nm using a microplate reader (BioTek, Winooski, VT, United States), and the Lucifer yellow concentration was calculated based on the standard curve. The results were expressed as the Lucifer yellow transmittance.

### Flow Cytometry Analysis

Intracellular calcium was detected by flow cytometry using the calcium-sensitive fluorescent probe Fluo-3/AM. Once the cells reached 80–90% confluence, the original culture medium was discarded and the cells were washed twice with PBS, followed by the addition of 0.25% trypsin (Solarbio, Beijing, China) to digest, and DMEM containing 10% fetal bovine serum to stop digestion. The cells were subsequently centrifuged at 60.82 × *g* for 5 min and then resuspended in PBS with the addition of Fluo-3/AM to a final concentration of 2.5 μM. The cell suspension was then incubated for 40 min at 37°C with shaking on an orbital shaker (Eppendorf, Germany). After incubation, the Fluo-3/AM-loaded cells were centrifuged again for 5 min, washed twice again, and then resuspended in PBS for subsequent pretreatment. The experimental groups were as above, and each group had three replicates. Cell suspensions were treated with various concentrations of HSYA (9, 27, and 81 μmol/L) or GB (10^-7^ μmol/L) and incubated for a further 30 min. PAF was then added to each tube to stimulate the cells and they were detected using a BD LSRFortessa (BD Biosciences, Franklin Lakes, NJ, United States) for 2 min. The mean fluorescence intensity (parent%) reflected the intracellular calcium ion concentration, at an excitation wavelength of 488 nm and an emission wavelength of 526 nm.

### Western Blot Analysis

Cells were washed twice with cold PBS (pH 7.4) and RIPA lysis buffer mixed with phosphatase inhibitor, and phenylmethanesulfonyl fluoride was then added to the cell samples, followed by centrifugation at 14,000 *g* for 15 min at 4°C to extract total proteins. Protein concentrations were measured using a bicinchoninic acid assay kit (Beyotime Institute of Biotechnology, Jiangsu, China), and protein samples were then separated by sodium dodecyl sulfate-polyacrylamide gel electrophoresis and transferred to nitrocellulose membranes. The membranes were blocked with 5% non-fat milk/Tris-buffered saline–Tween for 2 h, and then incubated with the following primary antibodies: PKC, phospho-IκB, phospho-ERK, phospho-JNK, phospho-p38, IκB, ERK, JNK, p38, and GAPDH (1:500–1000 dilution) overnight at 4°C. The membranes were subsequently washed three times with Tris-buffered saline–Tween and incubated with secondary IRDye^®^-conjugated goat anti-mouse or goat anti-rabbit antibodies (LI-COR Biosciences, Lincoln, NE, United States) at 1:8000 dilution for 50 min at room temperature. The membranes were washed a further three times, and the bands were scanned and their intensities measured using an Odyssey infrared imaging system (Gene Company, Beijing, China).

### Dual-Luciferase Reporter Gene Analysis

The transcriptional activities of NFκB and AP1 in HSAECs after PAF stimulation were measured by dual-luciferase reporter assay. The pNFκB-luc and pAP1-lcu reporter plasmids were produced by Beyotime Institute of Biotechnology. pAP1-luc was based on the pGL6 plasmid with multiple AP1-binding sites inserted at its multiple cloning site, and was used to detect the activation level of AP1 with high sensitivity. pNFκB-luc was a reporter gene plasmid used to detect the transcriptional activity levels of NFκB, with multiple NFκB-binding sites inserted at its multiple cloning sites in the pGL6 plasmid as a template. pRL-SV40 plasmid was chosen as an internal control. Cells were added into 24-well culture plates and cultured for 24 h to produce an 80–90% confluent monolayer. Cells in each well were then transfected with pNFκB-luc or pAP1-luc (500 ng) simultaneously with 50 ng of pRL-SV40, using Lipofectamine 3000 (Invitrogen). After 24 h, culture medium containing PAF with or without HSYA and/or GB was added to cells, followed by the addition of a passive lysis solution to produce cell lysates, and the cell lysates were harvested and detected in a 96-well plate. Cells were analyzed using the dual-luciferase reporter assay system (Promega, Madison, WI, United States) according to the manufacturer’s protocol. The relative light unit of firefly luciferase was normalized to that of *Renilla* to evaluate NFκB and AP1 activation levels.

### Statistical Analysis

Data were presented as mean ± standard deviation (SD). The significances of differences in various parameters between groups were analyzed using SPSS 13.0 (SPSS Inc., Chicago, IL, United States) by one-way analysis of variance (ANOVA) followed by Newman–Keuls *post hoc* test for multiple comparison tests. Figures were generated using GraphPad Prism 5.0 (GraphPad Software Inc., La Jolla, CA, United States) software. A *p* value < 0.05 was considered statistically significant.

## Results

### Effects of HSYA on IL-6, IL-1β, and TNF-α mRNA Expression in PAF-Stimulated HSAECs

**FIGURE 3:**
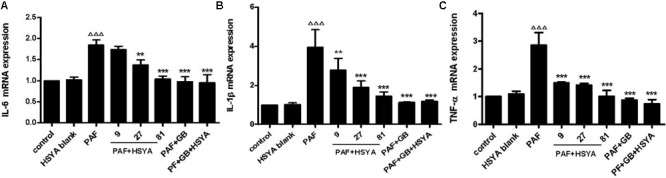
Effects of HSYA on IL-6 **(A)**, IL-1β **(B)**, and TNF-α **(C)** mRNA expression levels in PAF-stimulated HSAECs determined by RT-PCR. Data presented as mean ± SD, *n* = 3 per group. ANOVA, *p* < 0.0001 **(A)**, *p* < 0.001 **(B)**, *p* < 0.0001 **(C)**. *post hoc* ^ΔΔΔ^*p* < 0.001 versus control group, ^∗^*p* < 0.05, ^∗∗^*p* < 0.01, ^∗∗∗^*p* < 0.001 versus PAF group.

Bronchial epithelial cells produce large amounts of inflammatory factors during airway inflammation in asthma patients. We measured the effects of HSYA on IL-6, IL-1β, and TNF-α mRNA expression levels in PAF-stimulated HSAECs by RT-PCR. The gene expression levels of these factors were largely unaffected by HSYA, but were significantly increased by PAF (**Figure 3**). Expression levels were reduced by pretreatment with various concentrations of HSYA (9, 27, and 81 μmol/L) compared with the PAF group, in a concentration-dependent manner. mRNA levels of these cytokines were reduced almost to control levels in cells treated with PAF + GB and PAF + GB + HSYA. However, there was no significant difference between the PAF + GB and PAF + GB + HSYA groups. These results suggest that HSYA may exert a protective effects on PAF-induced mRNA expression in HSAECs.

### Effects of HSYA on IL-6, IL-1β, and TNF-α Protein Expression in PAF-Stimulated HSAECs

**FIGURE 4:**
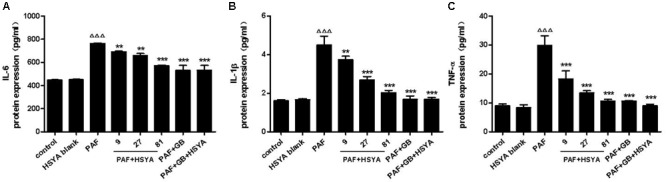
Effects of HSYA on IL-6 **(A)**, IL-1β **(B)**, and TNF-α **(C)** protein expression levels in PAF-stimulated HSAECs measured by ELISA. Data presented as mean ± SD, *n* = 3 per group. ANOVA, *p* < 0.0001 **(A)**, *p* < 0.0001 **(B)**, *p* < 0.0001 **(C)**. *post hoc* ^ΔΔΔ^*p* < 0.001 versus control group, ^∗∗^*p* < 0.01, ^∗∗∗^*p* < 0.001 versus PAF group.

We pretreated HSAECs with increasing concentrations of HSYA (9, 27, and 81 μmol/L) or GB (10^-7^ μmol/L)/GB + HSYA (81 μmol/L) for 30 min, followed by PAF stimulation for 12 h, and detected the protein levels of IL-6, IL-1β, and TNF-α in the supernatants by ELISA (**Figure 4**). Protein levels of these cytokines were significantly higher in the PAF-treated compared with the control cells. HSYA concentration-dependently decreased the production of IL-6, IL-1β, and TNF-α in the PAF-treated cells, with similar effects in the PAF + GB and PAF + GB + HSYA groups. There was no significant difference between the HSYA blank and normal control groups.

### Effect of HSYA on Monolayer Permeability in PAF-Stimulated HSAECs

**FIGURE 5:**
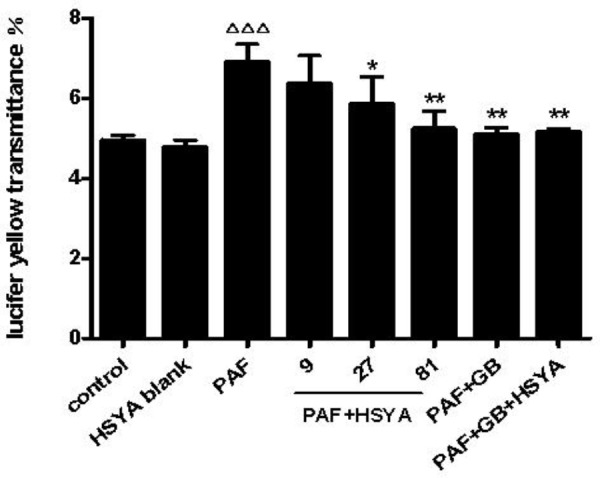
Effect of HSYA on transmittance of Lucifer yellow (indicating paracellular permeability) in PAF-stimulated HSAECs. Data presented as mean ± SD, *n* = 3 per group. ANOVA, *p* < 0.0001, *post hoc* ^ΔΔΔ^*p* < 0.001 versus control group, ^∗^*p* < 0.05, ^∗∗^*p* < 0.01 versus PAF group.

Airway epithelial barrier function is destroyed during early airway inflammation in asthmatic diseases, thus promoting development of the disease. In this study, we observed the changes in paracellular permeability of HSAECs in response to PAF, and the effects of HSYA on this process. The transmittance of Lucifer yellow, as an indicator of permeability of the epithelial monolayer barrier, was significantly higher in PAF-treated compared with normal control cells (**Figure 5**), while this was alleviated by HSYA in a concentration-dependent manner. GB had a similar effect, and this was also similar to the PAF + GB + HSYA group. There was no significant difference between the HSYA blank and control groups.

### Effects of HSYA on Cytosolic Calcium Level and PKC Protein Expression in PAF-Stimulated HSAECs

**FIGURE 6:**
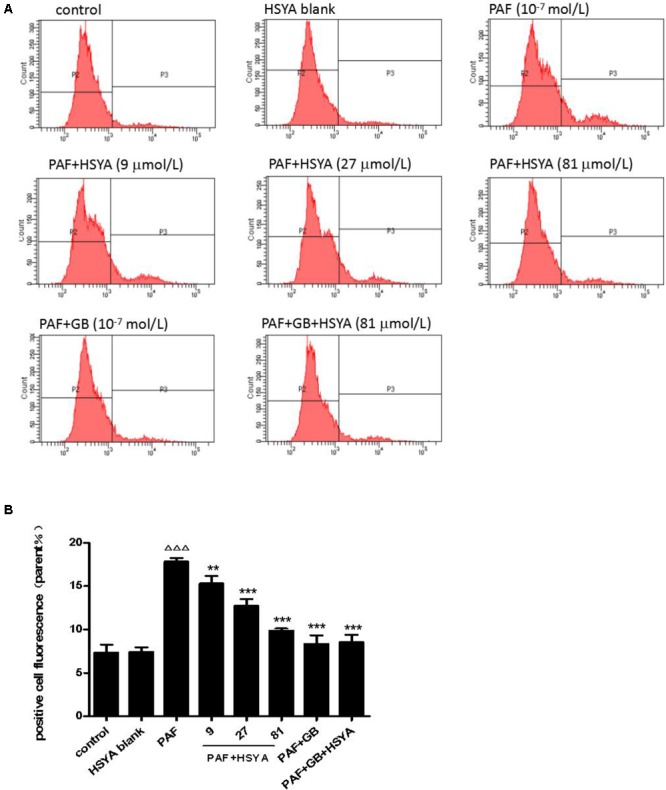
Effect of HSYA on intracellular calcium levels in PAF-stimulated HSAECs, detected by flow cytometry **(A, B)**. Data presented as mean ± SD, *n* = 3 per group. ANOVA, *p* < 0.0001, *post hoc* ^ΔΔΔ^*p* < 0.001 versus control group, ^∗∗^*p* < 0.01, ^∗∗∗^*p* < 0.001 versus PAF group.

We detected changes in intracellular calcium in HSAECs after PAF stimulation and evaluated the protective effect of HSYA. Intracellular calcium levels were similar in the HSYA blank and control groups, but levels were significantly increased by PAF, and this change could be alleviated by HSYA in a concentration-dependent manner (**Figure 6**). The damage was also attenuated by GB, with similar intracellular calcium levels in cells in the PAF + GB and PAF + GB + HSYA groups.

**FIGURE 7:**
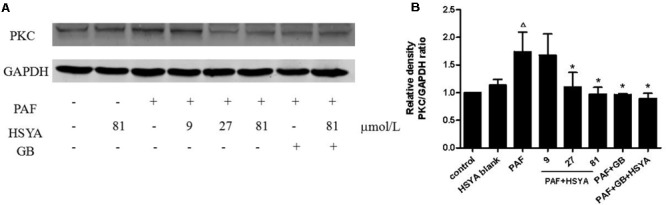
Effect of HSYA on protein expression level of PKC in PAF-stimulated HSAECs analyzed by western blotting **(A)**. Densitometric analyses of PKC **(B)**. Data presented as mean ± SD, *n* = 3 per group. ANOVA, *p* < 0.05, *post hoc* ^Δ^*p* < 0.05 versus control group, ^∗^*p* < 0.05 versus PAF group. GAPDH was used as an internal control.

PAF stimulation significantly up-regulated PKC protein expression levels, while HSYA itself had no effect on PKC levels in normal cells (**Figure 7**). The increased PKC levels could be reduced by 27 or 81 μmol/L HSYA and were also attenuated by GB, to a level similar to that in the PAF + GB + HSYA group.

### Effects of HSYA on MAPK (ERK, JNK, p38) Pathway and Transcriptional Activities of NFκB and AP1 in PAF-Stimulated HSAECs

**FIGURE 8:**
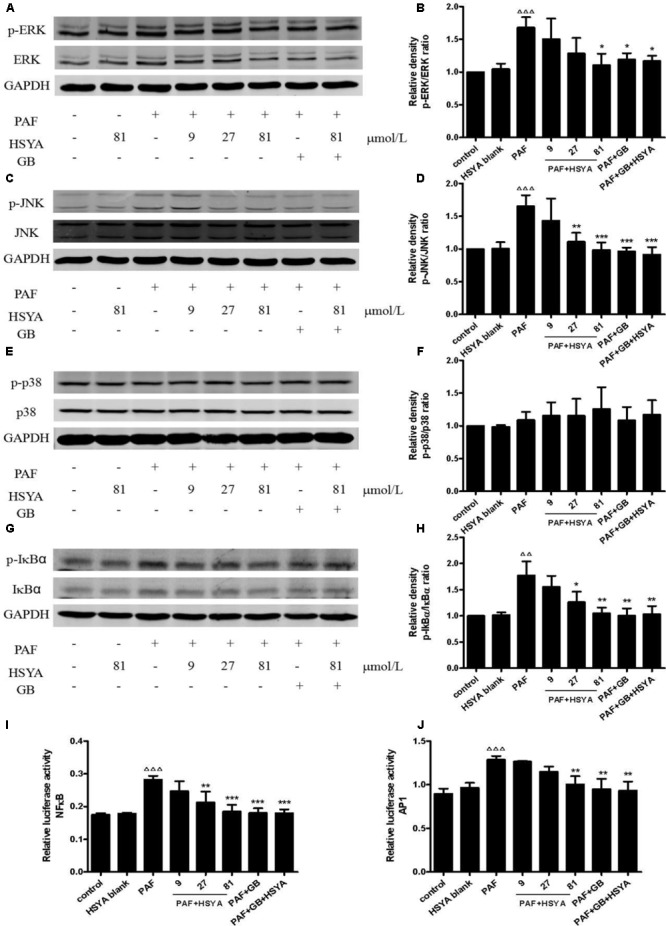
Effects of HSYA on MAPK (ERK, JNK, p38) pathway and transcriptional activities of NFκB and AP1 in PAF-stimulated HSAECs. Phosphorylation levels of ERK **(A)**, JNK **(C)**, p38 **(E)**, and IκB **(G)** were determined by western blot and quantified by densitometric analysis **(B, D, F, H)**. Binding activities of NFκB **(I)** and AP1 **(J)** were determined by dual-luciferase assay. Data presented as mean ± SD, *n* = 3 per group. ANOVA, *p* < 0.001 **(B)**, *p* < 0.001 **(D)**, *p* > 0.05 **(F)**, *p* < 0.01 **(H)**, *p* < 0.001 **(I)**, *p* < 0.001 **(J)**. *post hoc* ^ΔΔ^*p* < 0.01, ^ΔΔΔ^*p* < 0.001 versus control group, ^∗^*p* < 0.05, ^∗∗^*p* < 0.01, ^∗∗∗^*p* < 0.001 versus PAF group. GAPDH was used as an internal control.

We detected the phosphorylation levels of ERK, JNK, p38, and IκB by western blot, and the transcriptional activities of NFκB and AP1 by dual-luciferase reporter assay. Phospho-JNK and phospho-ERK levels in HSAECs were significantly increased in PAF-treated compared with control cells, and these increases were inhibited by HSYA, with similar results to the PAF + GB and PAF + GB + HSYA groups (**Figures 8A–D**). However, there was no significant difference between the PAF and normal control groups in terms of PAF-stimulated phospho-p38 levels, the no effect of HSYA on p38 phosphorylation (**Figures 8E,F**). Phospho-IκB levels and the binding activities of NFκB and AP1 were increased by PAF, and this increase could be attenuated by HSYA in a concentration-dependent manner, while luciferase activities recovered to near-normal levels in the PAF + GB and PAF + GB + HSYA groups (**Figures 8G–J**).

## Discussion

Asthma is a chronic inflammatory respiratory disease involving many cells and cytokines, characterized by clinical symptoms including chest tightness, shortness of breath, wheezing, and dry cough (Woolcock et al., 1987; Erle and Sheppard, 2014). The pathogenesis of asthma is complex, involving repeated inflammatory exacerbations of the lung epithelium, bronchial smooth muscle hyperreactivity, mucous production, and lung tissue remodeling, resulting in irreversible airway obstruction (Kuperman et al., 2002; Kato and Schleimer, 2007). The control of chronic inflammation is thus a basic and important part of asthma treatment.

Platelet activating factor is an important mediator involved in many inflammatory reactions (Kooij et al., 2012; Luna-Gomes et al., 2013), and its role in asthma is particularly prominent. It can be produced by several types of inflammatory cells and participates in the pathogenesis of bronchial asthma. PAF has been reported to cause direct bronchial obstruction in animals under experimental conditions, and belongs to a group of factors known to increase bronchial tree hyperreactivity in humans (Cuss et al., 1986; Pretolani and Vargaftig, 1993). Y-24180, GB, and other PAF antagonists can relieve asthma symptoms and improve respiratory function, indicating that PAF plays an important role in the pathological process of asthma with airway inflammation (Kasperska-Zajac et al., 2008a,b Chu et al., 2011).

Hydroxysafflor yellow A is a water-soluble monomeric constituent isolated from the traditional Chinese medicine *C. tinctorius* L., and is a major component of safflor yellow, which is in turn the main active component of *C. tinctorius* L. Safflor yellow injection, which comprises >90% HSYA, has been broadly used to treat coronary artery disease in China (Jin et al., 2013). Various researchers have also reported that HSYA inhibited lipopolysaccharide-induced or bleomycin-induced pulmonary inflammatory injury in mice (Sun et al., 2010; Wu et al., 2012) and pulmonary fibrosis activation (Pan et al., 2017), and it was also reported to alleviate pulmonary hypertension (Bai et al., 2012) and polymicrobial sepsis (Wang et al., 2017). However, although HSYA has been shown to relieve ovalbumin-induced asthma in mice (Piao et al., 2016), the potential mechanisms remain unclear. The current study thus provides the first evidence for the inhibition of PAF-induced asthma-related inflammatory activation in HSAECS by HSYA.

Airway epithelial cells represent the first barrier against environmental stimuli such as viruses, bacteria, and allergens. Normal airway epithelial cells are shielded from external allergens by tight junctions, but damage to the airway epithelium results in increased release of inflammatory mediators, and loss of barrier function, allowing the stimuli to reach the submucosa and cause smooth muscle spasm and AHR. Airway epithelial cells are thus important structural cells in the pathogenesis and development of asthma (Hammad and Lambrecht, 2015; Liu et al., 2018). As an important driver of inflammation in the bronchial epithelium (Howard, 2009), we showed that PAF stimulation increased the expression of IL-6, IL-1β, and TNF-α in HSAECs, and this effect was reduced by HSYA, thus potentially attenuating the secretion of inflammatory cytokines in asthma patients. Previous studies showed that PAF could disrupt the blood–brain barrier (Fang et al., 2014), enhance vascular endothelial cell permeability (Fang et al., 2011; Brailoiu et al., 2018), cause endothelial contraction, and increase capillary permeability (Palur Ramakrishnan et al., 2017). In the current experiment, PAF increased the paracellular permeability of the epithelial monolayer barrier, with implications for airway epithelium barrier function; however, HSYA reduced this enhanced permeability caused by PAF and thus protected HSAECs against damage to their barrier function.

The PAF receptor is a heptahelical G-protein-coupled receptor, and activation of this receptor on target cells induces the activation of multiple second messenger systems, such as phospholipase A2 and phospholipase C, which in turn activate PKC (Takano et al., 1994; Izumi and Shimizu, 1995), while the release of stored calcium ions leads to increased intracellular calcium concentrations (Brailoiu et al., 2018). MAPKs, including Erk1/2, p38, and JNK, can be activated by PAF stimulation in a wide range of cell types (Maruoka et al., 2000; Miike et al., 2000; Lukashova et al., 2001; McLaughlin et al., 2006). Kravchenko et al. (1995) showed that PAF activated NFκB binding activity in Chinese hamster ovary cells expressing the PAF receptor, and PAF was also capable of inducing the AP-1 signaling pathway in bronchial epithelial cells (LeVan et al., 1998). These PAF-mediated multiple signaling pathways and pathological changes then aggravate the symptoms of asthma. In this study, we demonstrated that HSYA antagonized PAF-induced changes in these pathways *in vitro*, by concentration-dependently reducing the increase in intracellular calcium and the overexpression of PKC, and inhibiting the phosphorylation of ERK1/2, JNK, and IκB, and suppressing the binding activities of NFκB and AP1, thereby blocking the PAF-induced inflammation as a potential mechanism and target for asthma treatment. However, HSYA did not interfere with p38 phosphorylation, possibly because PAF stimulation did not induce p38 phosphorylation in HSAECs and no inhibitory effect of HSYA was therefore observed, or because HSYA may exert divergent properties in different tissues or cell types. Further studies are therefore needed to determine if and how HSYA affects the p38 pathway. It is noteworthy that the anti-inflammatory effect exerted by HSYA through the inhibition of NFκB signaling is just like some other natural phenolic compounds such as chamomile extract, harpagophytum aqueous extract, graminex pollen and the mixture of crocus sativus, serenoa repens and pinus massoniana extracts, these materials can inhibit inflammatory injuries through NFκB signal pathway (Menghini et al., 2016; Chiavaroli et al., 2017; Locatelli et al., 2017, 2018).

In this study we used the PAF receptor inhibitor GB to interrupt the PAF-related signaling pathway and as a positive control drug. Both HSYA and GB exerted inhibitory effects on PAF-induced inflammatory activation in HSAECs, with GB and GB + HSYA (81 μmol/L) having similar effects. This suggests that HSYA and GB may act via the same pathway, and that the inhibitory effect of HSYA on PAF-induced activation of HSAECs may depend on the PAF receptor. HSYA may act on the cell surface, because its water-soluble properties mean that it cannot penetrate through the plasma membrane (Li et al., 2007). We therefore hypothesize that HSYA exerts its anti-inflammatory effects by blocking the interaction between PAF and the PAF receptor, thus suppressing its downstream signal transduction and blocking the related functional changes associated with asthma. However, further studies are needed to investigate this hypothesis.

In summary, this study demonstrated for the first time that HSYA could significantly inhibit PAF-induced inflammatory activation in HSAECs by inhibiting the PKC and MAPK signaling pathways and suppressing the activities of NFκB and AP1. These findings may partially explain the possible mechanism of HSYA in relieving asthma, and provide further evidence to support its potential use as an effective therapeutic agent in patients with bronchial asthma.

## Author Contributions

XG completed the experimental work and wrote the paper. MJ designed and supervised the experiments and revised the primary manuscript. BZ was responsible for analysis of HSYA. MZ and RP contributed to cell culture and partial molecular biological experiments.

## Conflict of Interest Statement

The authors declare that the research was conducted in the absence of any commercial or financial relationships that could be construed as a potential conflict of interest.
